# Phase I dose-escalation study of F50067, a humanized anti-CXCR4 monoclonal antibody alone and in combination with lenalidomide and low-dose dexamethasone, in relapsed or refractory multiple myeloma

**DOI:** 10.18632/oncotarget.25156

**Published:** 2018-05-08

**Authors:** Guillemette Fouquet, Stéphanie Guidez, Valentine Richez, Anne-Marie Stoppa, Christophe Le Tourneau, Margaret Macro, Cécile Gruchet, Arthur Bobin, Niels Moya, Thomas Syshenko, Florence Sabirou, Anthony Levy, Paul Franques, Hélène Gardeney, Lionel Karlin, Lotfi Benboubker, Monia Ouali, Jean-Claude Vedovato, Pierre Ferre, Mariya Pavlyuk, Michel Attal, Thierry Facon, Xavier Leleu

**Affiliations:** ^1^ Institut Imagine, Unité Inserm U1163, Centre National de la Recherche Scientifique CNRS ERL8254, Paris, France; ^2^ Hôpital La Milétrie, Centre Hospitalier Universitaire, Poitiers, France; ^3^ Institut Paoli Calmettes, Marseille, France; ^4^ Institut Curie, Paris, France; ^5^ Centre Hospitalier Universitaire, Caen, France; ^6^ Centre Hospitalier Lyon Sud, Lyon, France; ^7^ Centre Hospitalier Universitaire Tours, Tours, France; ^8^ Institut Claudius Regaud, Institut Universitaire du Cancer de Toulouse Oncopole, Toulouse, France; ^9^ Service des Maladies du Sang, Centre Hospitalier Régional Universitaire, Lille, France; ^10^ Institut de Recherche Pierre Fabre, Toulouse, France; ^11^ Inserm Centre d’Investigation Clinique U1402, Centre Hospitalier Universitaire, Poitiers, France

**Keywords:** multiple myeloma, CXCR4, monoclonal antibody, immunotherapy, homing of tumor cells

## Abstract

**Purpose:**

Multiple myeloma (MM) remains an incurable disease as tumor cells ultimately resist to all available drugs. Homing of tumor cells to the bone marrow microenvironment, involving especially the CXCR4/SDF-1 axis, allows them to survive, proliferate and resist to therapy. F50067, a humanized anti-CXCR4 IgG1 antibody, has promising preclinical activity in MM.

We present a phase I multicenter escalation study in relapsed/refractory MM (RRMM) to determine the maximum tolerated dose (MTD) for F50067 alone and in combination with lenalidomide and low dose dexamethasone (Len-Dex).

**Experimental design:**

14 end-stage RRMM patients received F50067 single agent (*n* = 10) or in combination with Len-Dex (*n* = 4).

**Results:**

One dose-limiting toxicity was observed, a grade 4 neutropenia lasting more than 7 days in combination arm. MTD could not be established. Thrombocytopenia was observed in 100% and neutropenia in 92.9% of patients with no cases of febrile neutropenia and no severe bleeding or hematoma. Non-hematological adverse events were of mild to moderate severity.

Nine patients (6 in single arm and 3 in combination arm) were evaluable for response, with 66.7% overall response rate (≥PR) in combination arm, and 33.3% of disease control (≥SD) in single agent arm. At the time of study termination, 55.6% had progressed.

**Conclusion:**

This study suggests that egression of tumor cells to the blood stream can represent a novel therapeutic strategy for MM. However, because of significant hematological toxicity, this study had to be discontinued. Further studies are needed to validate the feasibility of this approach in clinical practice.

## INTRODUCTION

Despite major therapeutic advances in recent years, multiple myeloma (MM) remains an incurable disease [1, 2], and all patients will ultimately progress with remission duration decreasing with each subsequent salvage regimen. Amongst mechanisms favoring resistance to drugs in relapsed/refractory MM (RRMM), the homing of tumor cells to the bone marrow microenvironment (BM) allows them to abnormally survive, proliferate and resist to therapy [3].

The CXCR4/SDF-1 axis has been found to play a major role in the BM localization of hematopoietic stem cells (HSC). A therapeutic CXCR4/SDF-1 axis disruption-based approach has already been validated with plerixafor, the first-in-class approved CXCR4 inhibitor. Plerixafor has indeed been used for years for its ability to mobilize HSC from BM to the blood stream where it can be harvested, in the setting of autologous transplantation in MM and lymphoma.

CXCR4 has also been shown to be expressed in many hematologic cancers as well as solid tumors and it was suggested to have a role in tumor survival [4]. The CXCR4/SDF-1 axis plays a major role in the BM localization of MM tumor cells [5] and in the regulation of MM cells trafficking [6, 7]. Preclinical models have therefore showed potential for alternative therapeutic uses for CXCR4 antagonists. *In vitro*, plerixafor inhibited SDF-1-induced migration and proliferation of a number of tumor cell lines [8]. Plerixafor has also been shown to induce disruption of the interaction of MM cells with the BM in MM animal models, resulting in mobilization of MM cells [7]. Moreover, plerixafor enhanced sensitivity of MM cells to multiple therapeutic agents *in vitro* and increased the tumor reduction induced by bortezomib *in vivo* [7]. A phase I study of plerixafor and bortezomib in RRMM patients [9] demonstrated transient de-adhesion of MM cells in most of the patients as soon as 2 hours post-plerixafor and during 4 to 24 hours. The combination of plerixafor and bortezomib was very active and generally well tolerated in this study.

F50067 (F50067 hz515H7-1), a humanized monoclonal IgG1 anti-CXCR4 antibody that specifically targets CXCR4, has demonstrated preclinical promising anti-tumor activity in MM. F50067 is expected to exert its effect through a dual role, disrupting the interaction of MM cells with the BM microenvironment and triggering both complement-dependent cytotoxicity (CDC) and antibody-dependent cellular cytotoxicity (ADCC). F50067 may also sensitize MM cells to the effects of lenalidomide and low-dose dexamethasone. A series of *in vivo* and *in vitro* preclinical investigations assessed that F50067 binds the human CXCR4, efficiently competes for SDF-1 binding, and inhibits CXCR4 receptor-mediated G-protein activation. F50067 antibody was shown to induce CDC and ADCC on a panel of cancer cells *in vitro*, with a percentage of cytotoxicity of around 40%. *In vivo*, F50067 displayed an anti-tumor activity in mouse xenograft models with multiple human tumor cell lines derived from Acute Myeloid Leukemia, B-cell lymphoma, Non Hodgkin T-cell lymphoma and MM. Rapid mobilization of white blood cells and CD34+ hematopoietic stem cells was observed in the peripheral circulation after a single F50067 administration in cynomolgus monkey, with a similar effect than the reference compound plerixafor.

The reported phase I dose escalation study was aimed to determine the maximum tolerated dose (MTD) for F50067 in monotherapy and in combination with lenalidomide and low dose dexamethasone in RRMM patients.

## RESULTS

### Patients’ characteristics

Overall, 14 patients with RRMM were enrolled to receive F50067 single agent (10 patients) or in combination with lenalidomide-dexamethasone (4 patients). Median age was 71 years, gender ratio M/F was 0.55. 5 patients were ISS (International Staging System) stage 3. The majority of patients had received a median of 6.6 (range 2–7) prior MM lines of therapy in F50067 single agent arm and 6 (5–7) in combination arm F50067 Len-Dex. All patients received IMiDs and proteasome inhibitor-based therapy as prior treatment: bortezomib (100%), lenalidomide (100%) and pomalidomide (respectively 75% in single agent arm and 90% in combination arm). Eleven patients had at least one autologous stem cell transplantation. Patients’ characteristics are described in Supplementary Table 1.

At the cut-off date, no patient remained under study treatment, a total of 36 evaluable cycles were administered (21 cycles in single agent arm and 15 cycles in combination arm) and the median number of cycles was 2 (1–8).

Among the 14 patients who received the study treatment all received a minimum of 4 consecutive doses of study treatment, except one patient in the F50067 single agent arm at dose level 0.1 mg/kg, who was therefore replaced for DLT assessment. The repartition for the 14 patients was as follows: Arm A: F50067 single agent, dose level 1 (DL1) = 1 patient, DL2 = 4 patients (only 3 evaluable for DLT), DL3 = 3 patients, DL4 = 2 patients included before study interruption; Arm B: F50067 Len-Dex arm, DL1 = 1 patient, DL2 = 3 patients.

### Efficacy analysis

Regarding the antitumor activity, 9 of the 14 patients enrolled were evaluable for response. Response assessments are presented in Table 1. The overall response rate (≥ partial response) was 66.7% (2/3 patients) in combination arm, and the clinical benefit rate (≥ minor response) was 33.3% (2/6 patients) in single agent arm. At the time of study termination, 5 patients had progressed (55.6%). In both patients with PR, the response was reached after second cycle of treatment. One of them had a response duration of 6 months.

**Table 1:** Response assessment (*n* = 9)

Dose of F50067 (mg/kg)	Single agent				Overall	F50067 Len-Dex		Overall
	0.03	0.1	0.3	1		0.03	0.1	
**N total of patients**	1	4	3	2	10	1	3	4
N response assessed^*^	0	2	3	1	6	1	2	3
**Partial response (PR)**	-	0	0	0	0	1	1	2 (66.7%)
**Stable disease (SD)**	-	0	1	1	2 (33.3%)	0	0	0
**ORR (≥PR)**	-	0	0	0	0	1	1	2 (66.7%)
**CBR (≥MR)**	-	0	0	0	0	1	1	2 (66.7%)
**Objective response (≥SD)**	-	0	1	1	2 (33.3%)	1	1	2 (66.7%)

^*^Patients not evaluable for response: no response at cycle 1, not evaluable at cycle 1 or not eligible

The overall survival (OS) and progression free survival (PFS) have not been estimated due to the small number of patients in single and combined arm (10 in the F50067 alone and 3 in F50067 Len-Dex) to avoid any misinterpretation. No extramedullary progression was reported.

### Safety analysis

Among all 14 treated patients, only one DLT was observed for the third patient treated in combination arm F50067 Len-Dex at dose level 2 (0.1 mg/kg): grade 4 neutropenia lasting more than 7 days occurring after the 4th dose of F50067. Safety results are presented in Table 2.

**Table 2:** DLT and adverse events, irrespective of their relationship to study drug (*n* = 14)

Dose of F50067 (mg/kg)	Single agent				F50067 Len-Dex	
	0.03	0.1	0.3	1	0.03	0.1
	*n* = 1	*n* = 4	*n* = 3	*n* = 2	*n* = 1	*n* = 3
**DLT (Dose-Limiting Toxicities)**						
**Neutrophils <0.5 × 10^9^/L for >7 days**	0	0	0	0	0	1
**Febrile neutropenia grade ≥3 in arm A and grade 4 in arm B**	0	0	0	0	0	0
**Platelets <25 × 10^9^/L for >7 days**	0	0	0	0	0	0
Any grade 3 non-hematological toxicity^*^	0	0	0	0	0	0
**Adverse events (AE)**						
**At least one AE,** all grades, *n*	1	4	3	2	1	3
**At least one AE,** grade 3–4, *n*	1	1	3	2	1	3
Hematological toxicities grade 3/4^**^						
**Anemia**	0	0	1	1	0	0
**Thrombocytopenia**	1	2	2	1	0	3
**Neutropenia**	1	3	2	1	0	3
**Febrile neutropenia**	0	0	0	0	0	0
**Non-hematological toxicities, grade 3–4**						
**Asthenia**	0	1	0	0	0	0
**Hyperhidrosis**	0	0	0	0	0	1
**Feeling cold**	0	0	0	0	0	1
**Pyrexia**	0	0	0	0	0	1
**Pneumonia influenza 1**	0	0	0	1	0	0
**Chest pain**	0	0	1	0	0	0
**Electrocardiogram QT prolonged**	0	0	0	0	0	1
**Acute coronary syndrome**	0	0	0	0	1	0
**Hypertension**	0	0	0	0	0	1
**Dyspnea**	0	0	0	0	1	0
**Pulmonary embolism**	0	0	0	0	1	0
**Femoral neck fracture**	0	0	0	0	1	0
**Hyponatremia**	0	0	0	0	1	0
**Acute renal failure**	1	0	0	0	0	0
**Oliguria**	0	0	1	0	0	0
**Anuria**	0	0	0	1	0	0
**Rectal hemorrhage**	1	0	0	0	0	0
**Cholestasis**	0	0	1	0	0	0
**Multi-organ failure**	0	0	0	1	0	0

^*^Any grade 3 non-haematological toxicity according to NCI CTCAE criteria (version 4.03) except unpremedicated nausea, vomiting, diarrhea or infusion reaction; and only in arm B grade 3 sensory neuropathy and grade 3 thromboembolic event.

^**^All hematological changes were reported as hematological toxicities but only 6 of them in 5 patients 3 in single and 2 in combination arm were reported as adverse events.

At baseline, most patients presented with grade 1/2 anemia, with a median hemoglobin level of 10.3 g/dL. Otherwise, consistent with the inclusion criteria, no significant cytopenia was observed. The median leucocyte level was 4.2 × 10^9^/L, the median neutrophil level was 2.49 × 10^9^/L and the median platelet level was 170 × 10^9^/L.

All treated patients displayed at least one hematological toxicity but only 6 of them in 5 patients were considered as adverse events (4 in single arm and 2 in combination arm). A decrease of the platelet count was reported for all 14 patients. Three patients reached a grade 3 and six a grade 4 thrombocytopenia, with a median nadir value of 43 × 10^9^/L in F50067 single agent and 53 × 10^9^/L in F50067 Len-Dex, and a minimal nadir value of 16 × 10^9^/L. Grade 3 or 4 thrombocytopenia recovery to ≥50 × 10^9^/L platelets occurred generally spontaneously within a few days after F50067 infusion and most often before the next infusion. In patients who responded to therapy, median time to platelet recovery was 7 days. For 2 patients platelet transfusions were required. No severe bleeding or hematoma was reported for any of these patients.

A decrease of the neutrophil count was reported for 13 out of 14 patients (92.9%). Grade 3 or 4 neutropenia was reported in 71.4% of patients (40% of grade 3 and 30% of grade 4 in single arm; 25% and 50% in combination in combination), and was usually transient (less than 24 hours). No episode of febrile neutropenia was reported.

Non hematological adverse events were generally of mild to moderate severity. Infusion reactions occurred in 3 patients in single arm treatment and were all grade 1–2. The most frequent non-hematological toxicities were asthenia with 5 out of 10 patients (50%) in single arm and 100% of patients in the combination arm and pyrexia in 7 of 10 patients (70%) in single arm and 2 of 4 (50%) in combination arm. The main non-hematological toxicities are described in Table 2.

Three serious adverse events were considered as related to F50067, in spite of the absence of compelling evidence, (i) in the F50067 Len-Dex arm: one patient experienced a grade 3 acute coronary syndrome and a grade 4 pulmonary embolism; (ii) in the single agent arm, one patient experienced a grade 2 cardiac failure.

Eleven deaths were reported in the study, 8/10 in single arm and 3/4 in F50067 Len-Dex arm. The cause of death was progressive disease for 9 patients, an influenza pneumopathy for one patient and an adverse event for the last patient (related to another study involving pomalidomide). Only one death was reported within 30 days after the drug discontinuation and was related to progressive disease. No death was considered drug-related by the investigators.

### F50067 dose reduction and treatment discontinuation

Only one patient had a dose reduction of F50067, in combination arm at dose level 0.03 mg/kg, because of an accidental removal of the infusion by the patient. One dose interruption was reported in single arm at day 1 of cycle 1 at dose level 0.3 mg/kg, for drug related non-hematological toxicity.

### Pharmacokinetics, immunogenicity and pharmacodynamics

**Figure 1:**
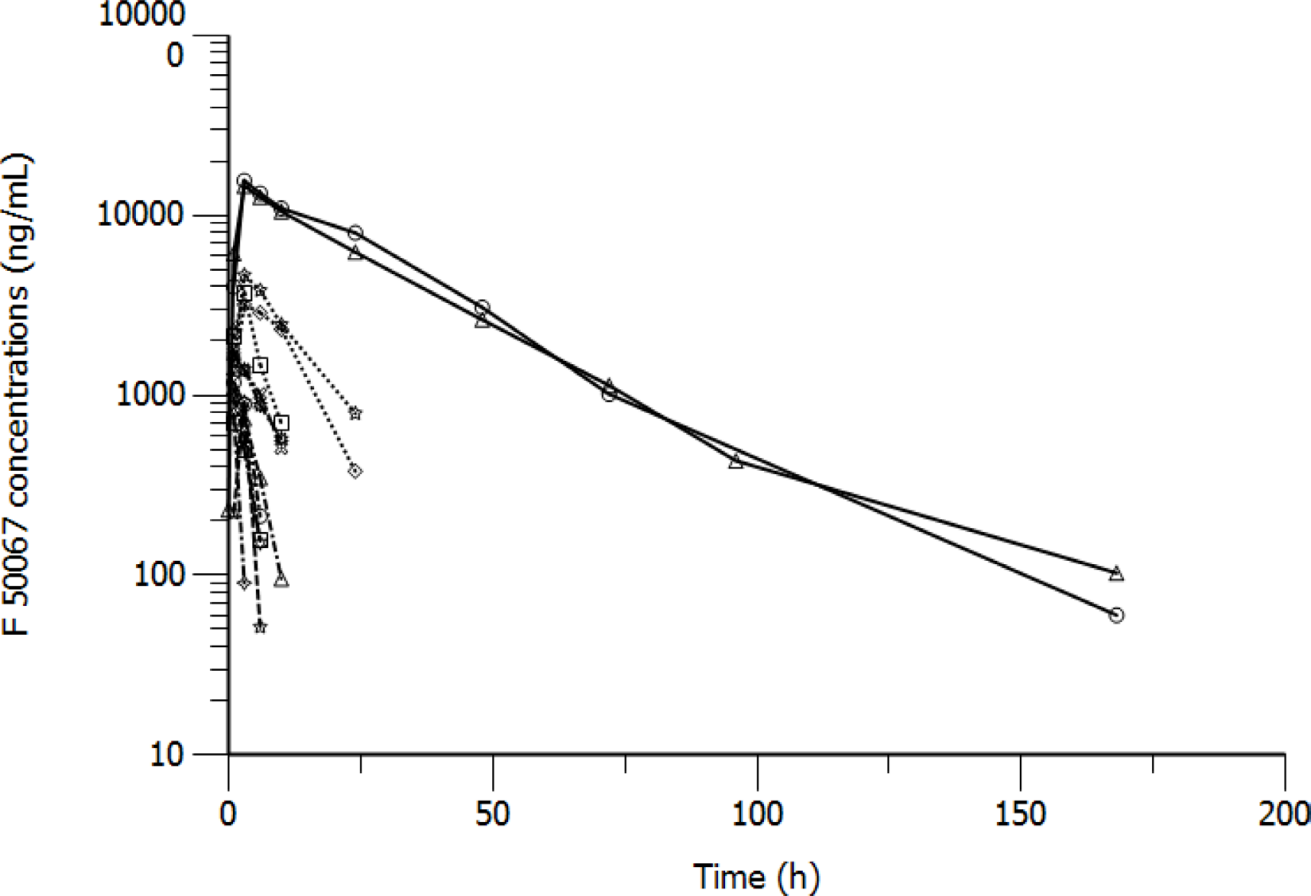
Pharmacokinetic profiles of patients treated with F50067 dosed intravenously at 0.03 mg/kg (dashed lines), 0.1 mg/kg (dashed dotted line), 0.3 mg/kg (dotted lines) and 1 mg/kg (solid lines)

**Figure 2:**
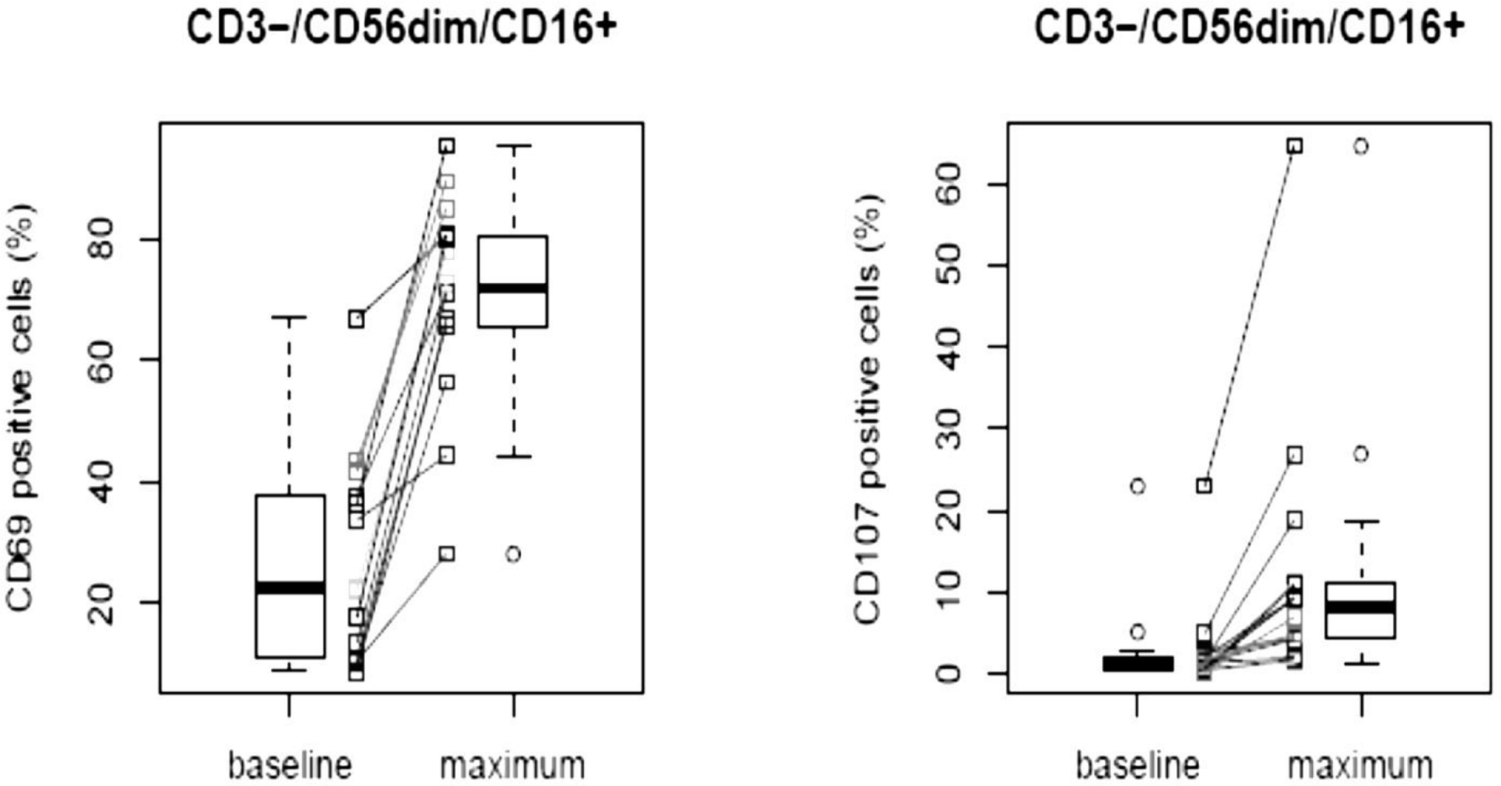
Activated circulating NK cells of patients before (baseline) and after treatment with F50067 administered intravenously at doses from 0.03 to 1 mg/kg “Maximum” value represents the maximal value of CD69 and CD107 positive cells observed after dosing of F50067.

Individual results for F50067 PK concentrations, immunogenicity assessment and biomarkers have been determined and reported. Pharmacokinetic profiles are reported on Figure 1. Both F50067 peak concentrations and also exposure duration above the lower limit of quantification increased with the administered dose. 102 samples from all patients were analysed for immunogenicity. 21 samples were confirmed as positive. Among them two patients had only one positive sample and one patient had 19 positive samples. This patient had pre-existing anti-drug antibodies with no major changes in titer after dosing with F50067. Among the exploratory pharmacodynamic biomarkers that were assessed, NK cell activation markers (CD69 and CD107a) were clearly increased after F50067 administration, at all dose levels, as shown on Figure 2.

## DISCUSSION

The CXCR4/SDF-1 axis plays an important role in many human diseases including HIV-1 infection, inflammatory diseases, and cancer. Overexpression of the CXCR4 receptor is a hallmark of many hematological malignancies such as acute myeloid leukemia (AML) [10], non Hodgkin lymphoma [11], chronic lymphocytic leukemia [12] or multiple myeloma [7] and usually correlates with invasiveness and poor prognosis [13]. The CXCR4/SDF-1 axis is indeed important for hematological tumor cell survival, migration and interaction with their protective microenvironment. The previous attempts to use CXCR4/SDF-1 axis disruption as a therapeutic tool appeared of interest and safe [14, 15].

Several agents targeting this axis have therefore been developed for clinical use especially in cancer [16–19]. CXCR4 inhibition appeared promising in *in vitro* MM models by enhancing the sensitivity of tumor cells to chemotherapy or other targeted therapies [7]. Pleriflaxor, a CXCR4 inhibitor, was used for chemosensitization in relapsed/refractory AML with encouraging results [10]. A phase I study of plerixafor and bortezomib was then conducted in RRMM patients, with a good efficacy and a favorable safety profile [9].

We have therefore sought to conduct a phase 1 study to assess F50067, a humanized anti-CXCR4 antibody, for safety and efficacy alone and in combination with lenalidomide and dexamethasone in RRMM patients. F50067 was of particular interest as it was expected to exert a dual efficacy mechanism. First, blocking the CXCR4/SDF-1 interaction would lead to egress of CXCR4-expressing tumor cells out of the tumor niche. In addition, F50067 was designed as an IgG1 monoclonal antibody, with Fc-mediated effector functions that would trigger both complement-dependent cytotoxicity (CDC) and antibody-dependent cellular cytotoxicity (ADCC) [20] against CXCR4 expressing tumor cells.

We report herein an overall response rate (ORR, ≥PR) of 66.7.% in combination arm and a clinical benefit rate (CBR, ≥MR) of 33.3% in monotherapy, which can be considered of interest in end stage RRMM. This study indeed included an elderly population with a median age of 71 years, and heavily pre-treated with 6.6 median prior lines of therapy. These results can, to a certain extent, validate the proof of concept that a disruption of the CXCR4/SDF-1 axis could be of interest in RRMM. The early signs of drug biological effect were evidenced by the activation of NK cells at all dose levels. This pharmacodynamic effect can be attributed to F50067 as the antibody was shown preclinical to have effector functions enabled [20]. This suggests that even if concentrations are not sustained due to rather low dose levels, the explored dose levels may be sufficient to drive to a therapeutic effect in patients. The observed results may warrant further drug and concept evaluation, providing that the hematological safety profile could be manageable.

Similar ADCC inducing effect has also been demonstrated as being key for the anti-tumor activity with elotuzumab, therapeutic antibody against CS-1 in multiple myeloma patients [21, 22].

This therapeutic approach has also been validated by others, with an ORR of 26% (including 13% of very good partial response (VGPR) or better) and a CBR of 32.6% reported in the phase I/II study combining pleriflaxor and bortezomib for RRMM patients [9]. More recently, another study combining a proteasome inhibitor with an inhibitor of the CXCR4/SDF-1 axis has been published [23]. In this phase IIa study, olaptesed pegol, a pegylated L-oligoribonucleotide that binds and neutralizes SDF-1 (also known as CXCL12), was administered to 28 RRMM patients in combination with bortezomib and dexamethasone. This study was aimed to prove the impact of a CXCL12 blockade in MM after preclinical [24] and phase I data [25]. The results were very promising with an ORR of 68% including 7% of complete response and 18% of VGPR, and a CBR of 75%. Thrombocytopenia (21% of grade 3–4) and anemia were the most frequent hematologic adverse events and were observed in almost 40% of patients [23]. Olaptesed alone was safe and well-tolerated, and no relevant additional toxicity was reported when combined with bortezomib and dexamethasone.

Unfortunately, we observed hematological toxicities at all F50067 doses in our study. Only one DLT was observed through the 5 dose levels and the MTD was not established. However, all patients who received F50067 indeed displayed hematological toxicities and non-hematological adverse events (AEs). 100% of patients experienced thrombocytopenia and 92.9% of neutropenia but only 2 grade 3 thrombocytopenia and 3 grade 3 neutropenia were reported as adverse events. Fortunately, these toxicities did not result in major complications with no cases of febrile neutropenia and no severe bleeding in our study, and there was therefore no need for specific treatment-related care. However, these observations suggest that patients treated with CXCR4 F50067 inhibitor therapy should be closely monitored for cytopenia. Management of these adverse events required red blood cells and platelets perfusion according to usual recommendations. These AEs could be expected in a certain extent, as hematopoietic stem cells (HSCs) and various cells of the hematopoietic lineage express CXCR4 and may be potential off targets for anti-CXCR4 antibodies [26]. Despite the absence of associated complications, the hematological toxicities were of concern as they resulted in a high risk to not reach an effective dose of F50067.

The pharmacokinetic assessment revealed that patients were exposed to F50067, increasing with dose levels as expected.

Interestingly, others CXCR4 inhibition-based approaches have shown that the safety profile of plerixafor was only satisfying for a short duration treatment. For instance in a study on 40 HIV-infected patients treated with plerixafor during 10 days by continuous intravenous infusions, many adverse effects were observed including diarrhea (48%), flatulence (43%), headache (40%), nausea (35%), abdominal pain (33%), abdominal distention (25%), tachycardia (25%), dizziness (25%), and paresthesias (23%) [21].

Despite the absence of significant activity with the F50067 CXCR4 inhibitor in this study, we believe that egression of tumor cells to the blood stream can still represent a novel therapeutic approach for MM. Inhibition of the CXCR4/SDF-1 axis seems to be a very promising approach, which could represent an important step forward if we manage to overcome the safety issues. Further studies are needed to improve the feasibility of this new therapeutic strategy in clinical practice.

## MATERIALS AND METHODS

### Patients

This is a multicenter, open-label, two-arm dose escalation phase I study of intra-venous (IV) humanized anti-CXCR4 monoclonal antibody (F50067) alone (single agent arm A) and in combination with oral lenalidomide and low dose dexamethasone (F50067-Len-Dex, combination arm B), in RRMM patients. Six IFM centers (Intergroupe Francophone du Myélome) participated in France, 4 have recruited patients for this study.

Eligible patients were at least 18 years old with confirmed RRMM and not eligible for or refusing stem cell transplantation after at least one but no more than 7 previous lines of treatment, including lenalidomide and bortezomib (or having a definitive contra-indication to bortezomib). Patients were also required to have a measurable disease, adequate bone marrow, renal and hepatic function. Patients primary refractory to lenalidomide (defined as never achieved a minimal response or better with lenalidomide) or not eligible for lenalidomide, with evidence of central nervous system (CNS) involvement and known active infection or other severe conditions, were excluded. All patients provided written informed consent, and the study protocol and amendment were approved by ethics committee.

The study was conducted in accordance with national regulations in France, and according to the Declaration of Helsinki.

### Procedures

F50067 was administered as a one-hour infusion on a weekly basis of a 28-day cycle. Lenalinomide was administered orally at 25 mg/day from day 1 to 21 of a 28 day-cycle. Dexamethasone was administered orally at 40 mg/week at days 1, 8, 15 and 22 of a 28 day-cycle.

Patients were planned to receive at least 2 cycles (4 to 8 doses of F50067) unless disease progression or unacceptable toxicity. Patients who tolerated the drug and showed no disease progression were allowed to continue treatment until untoward toxicity, progression of disease, choice of the patient or at the discretion of the responsible physician. Of note for patients who experienced a DLT during the first cycle, the study treatment was permanently discontinued.

Hematological toxicities were considered as AEs if they were serious, caused study discontinuation without any other symptom or caused treatment modification.

### Study design

This was initially a ping pong design between the 2 arms filling the single agent arm A before the combination arm B for each dose level. The dose-escalation scheme included up to 7 dose levels (DL) with F50067 at 0.03 mg/kg, 0.1 mg/kg, 0.3 mg/kg, 1 mg/kg, 3 mg/kg, 10 mg/kg and 20 mg/kg. The study planned the inclusion of 1 patient per cohort at dose level 1 (DL1) and 3 patients per cohort from DL2 onwards (unless dose-limiting toxicities **(**DLTs) were observed in the first 2 patients prior to enrolment of a third patient).

This design was modified by amendment on the review of safety data available on the first patients in order to perform the full dose escalation of F50067 as single agent arm A prior to testing the combination arm B, F50067-Len-Dex. Following completion of recruitment in combination arm at DL2 (0.1 mg/kg), the dose escalation was performed in single agent arm starting at DL3 (0.3 mg/kg) up to the maximum tolerated dose (MTD) or maximum planned dose (MPD) for F60067 as single agent.

### Endpoints (or objectives)

The primary endpoint was to determine the MTD of weekly or every-two-weeks IV F50067 up to a maximum planned dose (MPD) of 20 mg/kg in RRMM, alone and F50067-Len-Dex.

MTD was defined as the dose level at which at least 2 out of 3 or 2 out of 6 patients developed a DLT during the first cycle.

DLT was graded according to NCI CTC for adverse events (version 4.03) and was defined as one of the following drug-related adverse event occurring during the first cycle: (i) Neutrophils < 0.5 × 10^9^/L for >7 days, (ii) Febrile neutropenia grade ≥3 in arm A and grade 4 in arm B, (iii) Platelets < 25 × 10^9^/L for >7 days, (iiii) Any grade ≥3 non-hematological toxicity according to NCI CTCAE criteria, except unpremedicated nausea, vomiting, diarrhea or infusion reaction; and only in arm B grade 3 sensory neuropathy and grade 3 thromboembolic event.

Secondary Objective was to determine the following in patients with RRMM: the pharmacokinetic profile, pharmacodynamics, immunogenicity, and tumor response rate according to the International Myeloma Working Group (IMWG) criteria [10, 11] of F50067 alone and F50067-Len-Dex. All patients who received at least one dose of F50067 and for whom efficacy assessment after the first dose was available were included in the efficacy analysis.

### Safety analysis was done continuously on all treated patients

Safety was assessed by physical examination, complete blood cell count, serum chemistry and electrocardiogram at several time points. DLT and other adverse events were reported according to NCI CTCAE (version 4.03). For patients who experienced toxicity, relationship with exposure to F50067 was investigated.

### Pharmacokinetics and exploratory biomarkers

Blood and serum samples were collected at several time points to evaluate the pharmacokinetic profile, exploratory pharmacodynamics markers, presence of anti-drug antibodies and cell mobilization induced by F50067.

## CONCLUSIONS

This phase I study was aimed to establish the maximal tolerated dose of F50067, a CXCR4 inhibitor, as single agent and in combination with lenalidomide and low dose dexamethasone in RRMM patients. However, the sponsor decided to interrupt the study as no convincing activity was observed. The hematological toxicity and especially the thrombocytopenia were of concern in this study, with a high risk to not reach an effective dose, and a negative benefit risk balance.

## SUPPLEMENTARY MATERIALS TABLE




