# Supplementary material for: Phase I dose-escalation study of F50067, a humanized anti-CXCR4 monoclonal antibody alone and in combination with lenalidomide and low-dose dexamethasone, in relapsed or refractory multiple myeloma
Source: Oncotarget. 2018 May 8;9(35):23890–9. doi: 10.18632/oncotarget.25156 (PMC5963612; doi:10.18632/oncotarget.25156)
Supplement: Supplementary file 1 [file oncotarget-09-23890-s001.pdf]

Phase I dose-escalation study of F50067, a humanized anti-CXCR4 monoclonal antibody alone and in combination with Lenalidomide and low-dose dexamethasone, in relapsed or refractory multiple myeloma

SUPPLEMENTARY MATERIALS

Supplementary Table 1: Patients' characteristics by arm and dose level (*n* (%) unless specified). See Supplementary_Table_1
