# Supplementary material for: Phase I dose-escalation study of F50067, a humanized anti-CXCR4 monoclonal antibody alone and in combination with lenalidomide and low-dose dexamethasone, in relapsed or refractory multiple myeloma
Source: Oncotarget. 2018 May 8;9(35):23890–9. doi: 10.18632/oncotarget.25156 (PMC5963612; doi:10.18632/oncotarget.25156)
Supplement: Supplementary file 2 [file oncotarget-09-23890-s002.docx]

**Supplementary Table 1: Patients’ characteristics by arm and dose level (n (%) unless specified)**

| **Dose of F50067 (mg/kg)** | **Single agent** | | | | | | | | **F50067 Len-Dex** | | **Overall** |
| --- | --- | --- | --- | --- | --- | --- | --- | --- | --- | --- | --- |
|  | **0.03** | | **0.1** | | **0.3** | **1** | | | **0.03** | **0.1** |  |
| **N** | **n=1** | | **n=4** | | **n=3** | **n=2** | | | **n=1** | **n=4** | **n=14** |
| **Age at study entry, years** | | | | | | | | | | | |
| **Median** | 67 | 68 | | 58 | | | 61.5 | 74 | | 71 | 71 |
| **Min;Max** | 67;67 | 63;81 | | 57;62 | | | 57;66 | 74;74 | | 64;74 | 57;81 |
| **< 60 years, n (%)** | 0 | 0 | | 2 | | | 1 | 0 | | 0 | 3 (21.4) |
| **≥ 60 years, n (%)** | 1 | 4 | | 1 | | | 1 | 1 | | 3 | 11 (78.6) |
| **WHO performance status at study entry** | | | | | | | | | | | |
| **1, n (%)** | 1 | 3 | | 2 | | | 1 | 1 | | 3 | 11 (78.6) |
| **2, n (%)** | 0 | 1 | | 0 | | | 0 | 0 | | 0 | 1 (7.1) |
| **M protein Isotype of interest** | | | | | | | | | | | |
| **IgA, n (%)** | 0 | 1 | | 0 | | | 1 | 0 | | 1 | 3 (21.4) |
| **Free Light Chain, n (%)** | 0 | 1 | | 1 | | | 0 | 0 | | 1 | 3 (21.4) |
| **Beta-2-microglobulin at diagnosis (mg/L)** | | | | | | | | | | | |
| **Median** | NA | 4.25 | | 2.80 | | | NA | 2.70 | | 16.50 | 3.53 |
| **ISS at diagnosis** | | | | | | | | | | | |
| **III, n (%)** | 1 | 1 | | 0 | | | 1 | 0 | | 2 | 5 (35.7) |
| **Unknown, n (%)** | 0 | 2 | | 2 | | | 1 | 0 | | 1 | 6 (42.8) |
| **Presence of soft tissue plasmacytoma at study entry** | | | | | | | | | | | |
| **Yes, n (%)** | 0 | 1 | | 0 | | | 0 | 0 | | 0 | 1 (7.1) |
| **FISH results at diagnosis and/or study entry** | | | | | | | | | | | |
| **Not done, n (%)** | 1 | 3 | | 2 | | | 2 | 1 | | 3 | 12 (85.7) |
| **Del(17p), n (%)** | 0 | 1 | | 0 | | | 0 | 0 | | 0 | 1 (7.1) |
| **t(4;14), n (%)** | 0 | 0 | | 1 | | | 0 | 0 | | 0 | 1 (7.1) |
| **Interval from diagnosis to study entry (months)** | | | | | | | | | | | |
| **Median** | 60.6 | 60.5 | | 87.9 | | | 69.7 | 106.9 | | 85.3 | 77.5 |
| **Min;Max** | 61;61 | 28;126 | | 50;100 | | | 62;77 | 107;107 | | 83;132 | 28;132 |
| **Interval from diagnosis to first relapse (months)** | | | | | | | | | | | |
| **Median** | 33.9 | 15.5 | | 31.5 | | | 23 | 10.1 | | 45 | 27.25 |
| **Min;max** | 34;34 | 8;23 | | 28;35 | | | 21;25 | 10;10 | | 31;49 | 8;49 |
| **Number of lines of prior therapy, n** | | | | | | | | | | | |
| **Median** | 5 | 7 | | 5 | | | 6 | 5 | | 7 | 6 |
| **Prior therapies, n (%)** | | | | | | | | | | | |
| **Glucocorticoids** | 1 | 4 | | 3 | | | 2 | 1 | | 3 | 14 (100) |
| **Doxorubicin** | 0 | 2 | | 0 | | | 0 | 0 | | 1 | 3 (21.4) |
| **Cyclophosphamide** | 0 | 4 | | 3 | | | 1 | 0 | | 2 | 10 (71.4) |
| **Melphalan** | 1 | 3 | | 0 | | | 0 | 1 | | 2 | 7 (50) |
| **Bortezomib** | 1 | 4 | | 3 | | | 2 | 1 | | 3 | 14 (100) |
| **Carfilzomib** | 0 | 0 | | 0 | | | 0 | 0 | | 1 | 1 (7.1) |
| **Thalidomide** | 0 | 4 | | 1 | | | 0 | 1 | | 1 | 7 (50) |
| **Lenalidomide** | 1 | 4 | | 3 | | | 2 | 1 | | 3 | 14 (100) |
| **Pomalidomide** | 1 | 3 | | 3 | | | 2 | 1 | | 2 | 12 (85.7) |
| **Panobinostat** | 0 | 0 | | 0 | | | 0 | 1 | | 0 | 1 (7.1) |
| **Cisplatin** | 0 | 0 | | 1 | | | 1 | 0 | | 1 | 3 (21.4) |
| **Etoposide** | 0 | 0 | | 1 | | | 1 | 0 | | 1 | 3 (21.4) |
| **Vincristine** | 0 | 0 | | 0 | | | 0 | 0 | | 1 | 1 (7.1) |
| **Bendamustine** | 1 | 3 | | 2 | | | 2 | 0 | | 2 | 10 (71.4) |

*FLC: free light chains.NA: not available*

**Glucocorticoïds: dexamethasone, methylprednisolone, prednisone*
